# Clinicopathological Phenotype and Genetics of X-Linked Dystonia–Parkinsonism (XDP; DYT3; Lubag)

**DOI:** 10.3390/brainsci7070072

**Published:** 2017-06-26

**Authors:** Toshitaka Kawarai, Ryoma Morigaki, Ryuji Kaji, Satoshi Goto

**Affiliations:** 1 Department of Clinical Neuroscience, Institute of Biomedical Sciences, Graduate School of Medical Sciences, Tokushima University, Tokushima 770-8503, Japan; tkawarai@tokushima-u.ac.jp (T.K.); rkaji@tokushima-u.ac.jp (R.K.); 2 Parkinson’s Disease and Dystonia Research Center, Tokushima University Hospital, Tokushima 770-8503, Japan; morigakiryoma@hotmail.com; 3 Department of Neurodegenerative Disorders Research, Institute of Biomedical Sciences, Graduate School of Medical Sciences, Tokushima University, Tokushima 770-8503, Japan; 4 Department of Neurosurgery, Institute of Biomedical Sciences, Graduate School of Medical Sciences, Tokushima University, Tokushima 770-8503, Japan

**Keywords:** X-linked dystonia–parkinsonism, striatum, striosome, neurodegeneration, genetics, pathophysiology

## Abstract

X-linked dystonia–parkinsonism (XDP; OMIM314250), also referred to as DYT3 dystonia or “Lubag” disease, was first described as an endemic disease in the Philippine island of Panay. XDP is an adult-onset movement disorder characterized by progressive and severe dystonia followed by overt parkinsonism in the later years of life. Among the primary monogenic dystonias, XDP has been identified as a transcriptional dysregulation syndrome with impaired expression of the *TAF1* (TATA box-binding protein associated factor 1) gene, which is a critical component of the cellular transcription machinery. The major neuropathology of XDP is progressive neuronal loss in the neostriatum (i.e., the caudate nucleus and putamen). XDP may be used as a human disease model to elucidate the pathomechanisms by which striatal neurodegeneration leads to dystonia symptoms. In this article, we introduce recent advances in the understanding of the interplay between pathophysiology and genetics in XDP.

## 1. Introduction

X-linked dystonia–parkinsonism (XDP; OMIM314250), also known as DYT3 dystonia or “Lubag” disease, is a hereditary neurodegenerative disorder that primarily involves the striatum. XDP may be a unique human disease model used to investigate the pathogeneses of dystonia and parkinsonism. It was first described as an endemic disease on the island of Panay in the Philippines [1]. XDP is now classified as a primary monogenic torsion dystonia [2]. This adult-onset movement disorder is clinically characterized by progressive and severe dystonia, followed by overt parkinsonism in the later years of life [3]. Like Huntington’s disease (HD) and spinocerebellar ataxia 17 (SCA17), XDP has been identified as a transcriptional dysregulation syndrome [4]. XDP involves the impaired expression of the *TAF1* (TATA box-binding protein associated factor 1) gene [5], which is ubiquitously expressed and is an essential component of the transcription machinery [6]. The major neuropathology of XDP is progressive neuronal loss in the neostriatum (i.e., the putamen and caudate nucleus) [7,8,9], where compartmental and cell type-specific degeneration of striatal neurons is found [8,9]. Among the primary monogenic dystonias, XDP is a unique heredodegenerative disorder whose causative gene and functional pathology have been explicated. Here, we introduce recent advances in the understanding of the genetic and clinicopathological features of XDP.

## 2. Clinical Phenotype

XDP is originally endemic to the island of Panay in the Philippines [1]. As of 2001, its prevalence rate for the entire Philippines was 0.31 per 100,000, although it was as high as 5.24 per 100,000 in the island of Panay [10]. Currently, affected Filipinos with XDP are also found in the USA, Canada, Germany, UK, and Japan [3,11].

The striking clinical feature in patients with XDP is the contradictory biphasic movement disorder manifestation, namely dystonia and parkinsonism. The onset of the disease usually occurs in the late 30s to early 40s (mean, 39.67; range, 12–64) [3]. It usually starts with focal dystonia (94.3%), although some patients first present with parkinsonism (5.7%) [3]. On average, focal dystonia becomes generalized within four years (range, 1–23 years), and most patients (84%) present with generalized dystonia within five years of disease onset [3]. Eventually, the vast majority of patients with focal dystonia (97%) develop generalized dystonia [3]. Generalized dystonia affects the craniofacial and cervical segments, the upper and lower limbs, and the trunk [3,10]. Dystonia is typically predominant until the seventh year of illness. Parkinsonism becomes gradually more prominent between the seventh and 10th year after onset [11]. The mean duration from onset to predominant parkinsonism is 14 years (range, 7–25) years [3]. To date, 14 affected female patients have been reported, while more than 500 men have been diagnosed with XDP [12]. Homozygosity and skewed X-inactivation can lead to XDP in women [5,12]. Female XDP carriers are mostly asymptomatic and symptomatic female carriers usually manifest non-progressive focal dystonia, chorea, focal tremor, or non-progressive mild parkinsonism [12,13,14]. Pain is frequently observed in 80% of individuals with oromandibular dystonia and in 75% of those with truncal–axial dystonia due largely to muscle contraction [15].

Cognitive impairment in patients with XDP is usually mild. However, 76% of patients with XDP fail one of the following tests: the Mini-Mental State Examination, the Clock Drawing Test, and Frontal Assessment Battery [16]. There may be impairments in the dorsolateral frontal lobe functions of abstract thinking and conceptualization [16]. Executive dysfunction has been noted in three case reports [17,18,19]. Using a flanker task, a set of response inhibition tests used to assess the ability to inhibit inappropriate responses in a particular context, Beste et al. [20] have reported that error-related behavioral adaptation is markedly impaired in patients with XDP in the early disease stages. Abnormal error processing is correlated with dystonia severity, but not with parkinsonism [20]. Interestingly, response inhibition in a go/no-go task and corresponding electroencephalograms are normal in patients with XDP [20]. This indicates a lack of impulsivity. The authors concluded that dysfunction in behavioral adaptation might reflect striosomal dysfunction [20]. The same group reported faster and more accurate perceptual decision-making performance in predictive coding processes using distracting pitches of tones in patients with XDP when compared to healthy controls [21]. Predictive coding is the ability to predict upcoming events on the basis of extracted regularities of previous inputs, and is known to depend on fronto-striatal circuits [22]. There was less response slowing and a smaller reduction of accuracy when XDP patients, in their early dystonic stages, were confronted with the distracting pitches of the tones compared to controls [21]. These results suggested that striosomal dysfunction was associated with this paradoxical benefit for perceptual decision-making processes [21]. The authors hypothesized that striosomal dysfunction in XDP patients reduced its interferences to the matrix, which might result in better perceptual decision-making processes [21].

The mean age of death in patients with XDP is 55.59 (range, 31–81) years. Death usually occurs due to malnutrition or aspiration pneumonia [3,11]. Causes of death include a non-ignorable number of suicides (9%) [3]. Notably, 14.3% of patients with XDP have major depression, according to the Diagnostic and Statistical Manual-IV (DSM-IV) criteria [23]. In addition, 35.7% of patients with XDP have anxiety disorders, including social phobia (28.6%), agoraphobia (21.4%), and panic disorder (7.1%), as determined using the DSM-IV [23]. Depressive symptoms are highly prevalent in up to 92.9% of patients, as determined using the Zung Self-Rating Depression Scale, and in 54.8% of patients when they are assessed using the Hamilton Depression Rating Scale (HAM-D) [16,23]. A worsening of both Burke–Fahn–Marsden Dystonia Rating Scale (BFM-DRS) and Schwab and England Activities of Daily Living scores are significantly correlated with positive HAM-D scores [16]. The functional impairment caused by severe dystonia or end-stage parkinsonism usually induces severe disability in activities of daily living (ADL) [3]. Thus, both the neuropathological changes in the central nervous system and disability in ADL may affect mood in patients with XDP, which might explain the high rate of suicide. 

## 3. Neuroimaging

A review of magnetic resonance imaging films from 46 patients with XDP by Pasco et al. indicated six of the patients had normal findings, while 40 had abnormal findings [24]. All six films without abnormal findings were imaged during the early dystonic phase. All of the 40 films with abnormal findings contained hyperintense putaminal rims and 72% displayed atrophy of the caudate head. Putaminal atrophy was found in 30% of the films, which were imaged mostly during the later parkinsonism stage.

Severe bilateral putaminal metabolic deficits assessed using [^18^F] fluorodeoxyglucose positron emission tomography (PET) have been reported from the early to late stages of XDP [7,25,26]. Using [^18^F] fluorodopa, normal and reduced dopamine re-uptake by presynaptic terminals have been observed in patients with the early dystonic stage and the moderately severe parkinsonism stage, respectively [7,25,26].

Dopamine transporter imaging using [^123^I] β-carbomethoxy-iodophenyl-nortropane and [^123^I] fluoropropyl-2-beta-carbomethoxy-3-beta(4-iodophenyl) nortropane single-photon emission computed tomography (SPECT) studies show moderately reduced uptake in the bilateral putamen in patients with XDP in the early to middle stages of the disease [14,27,28]. There is an inconsistency between PET and SPECT studies related to presynaptic dopaminergic function in XDP patients in the early dystonia phase. While [^18^F] fluorodopa PET studies showed normal presynaptic dopaminergic terminal innervation, dopamine transporter imaging SPECT studies demonstrated reduced presynaptic dopaminergic afferents [7,25,26,27,28]. Dopamine D2 receptor (D_2_R) imaging using [^123^I] iodobenzamide (IBZM)-SPECT indicates a slight to moderate decrease in D_2_R expression in the bilateral striatum in patients with XDP in the early to middle stages of disease [27,28]. Longer disease duration is correlated with lower IBZM-binding ratios in the right striatum [28]. However, IBZM-SPECT cannot distinguish pre-synaptic D_2_R from post-synaptic D_2_R, thus it cannot be judged whether IBZM-SPECT reduction reflects striatal neuron loss or the loss of D_2_R-positive afferents [29]. It is difficult to derive any definitive conclusion from these studies due to small sample size. Walter et al. enrolled 90 Filipino participants in a brain sonography study [30]. They reported presynaptic alteration in XDP patients with prominent parkinsonism by showing a positive correlation between hyperechogenicity in substantia nigra and the severity of parkinsonism. On the other hand, there was a negative correlation between hyperechogenicity in substantia nigra and the severity of dystonia. A majority of XDP patients present pure dystonia or a dystonia-predominant phenotype from the early stages, suggestive of a predominant involvement of postsynaptic dysfunction [14]. Few XDP patients represent pure parkinsonism, with a subsequent manifestation of dystonia in later stages or a parkinsonism-predominant phenotype from the early stages suggestive of a predominant involvement of presynaptic defect [14]. XDP-related genetic changes may underlie the genesis of these different phenotypes [14,30].

## 4. Treatments

A variety of pharmacological and surgical therapies have been proposed for the treatment of dystonia and parkinsonism in patients with XDP. Oral medications such as benzodiazepines, anticholinergic agents, antipsychotic agents, and anti-parkinsonian medications have failed to show convincing benefit [11]. Zolpidem and tetrabenazine might be effective for some individuals with advanced dystonia [14,31,32]. Levodopa and dopamine agonists might have some beneficial effects, particularly in individuals with pure parkinsonism [14]. Long-term treatment with L-3-(3,4-Dihydroxyphenyl)alanine (L-DOPA) does not induce L-DOPA-induced dyskinesia [14].

Chemodenervation using muscle afferent block and botulinum toxin type A (BoNT-A) have been used in patients with XDP, especially during the focal, multifocal, and segmental stages of dystonia [11,15]. It was also noted that in 109 patients with XDP, BoNT-A injections produced significant improvements in oromandibular, lingual, and truncal-axial dystonias, with a marked reduction in associated pain [15]. 

Deep brain stimulation of the globus pallidus internus (GPi-DBS) has been used in several patients with XDP and severe generalized dystonia [18,33,34,35,36,37,38,39]. The effects of bilateral GPi-DBS are always immediate and striking [38] and lead to a 34–95% improvement in dystonia symptoms, as determined using BFM-DRS [18,33,34,35,36,37,38,39]. In general, GPi-DBS may have beneficial effects on dystonia symptoms in patients with XDP, but not on parkinsonian symptoms [35]. Notably, a study using intraoperative pallidal microdialysis suggested that GPi-DBS might suppress dystonia symptoms by enhancing local gamma-aminobutyric acid (GABA) release in the GPi [39]. Another study of intraoperative local field potentials also provides evidence suggesting that GPi-DBS might improve dystonia symptoms by suppressing beta hypersynchrony in the GPi [37].

## 5. Genetics

**Figure 1:**
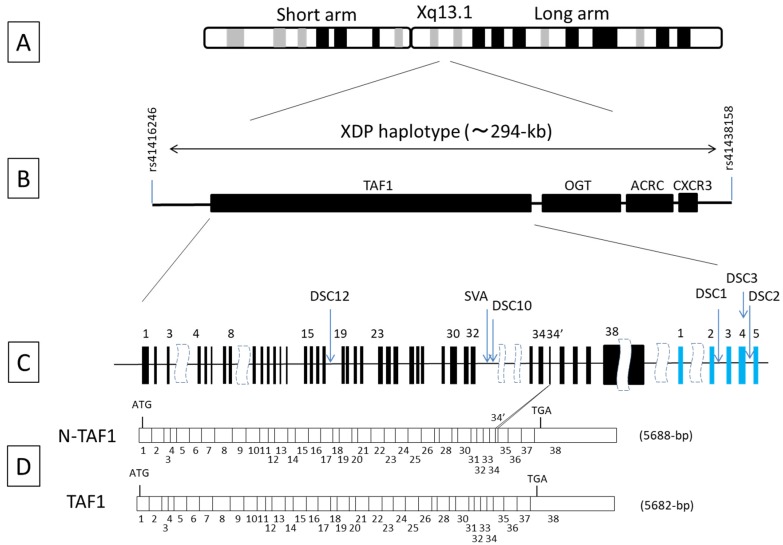
Scheme of X-linked dystonia–parkinsonism (XDP) locus, candidate genes, genomic organization of *TAF1*, approximate position of nucleotide variants, and *TAF1* transcript reduced in an XDP patient. (**A**) Diagram of human X chromosome. The XDP locus has been mapped to the chromosomal region Xq13.1 at the long arm of the X chromosome; (**B**) Physical and genetic map at the XDP locus. Whole genome sequencing and linkage disequilibrium analysis narrowed the XDP haplotype to approximately a 294 kb region flanked by the two single nucleotide polymorphism, rs41416246 and rs41438158. Four genes are mapped to the refined XDP locus: TATA-box binding protein associated factor 1 (*TAF1*), *O*-linked *N*-acetylglucosamine (GlcNAc) transferase (*OGT*), acidic repeat-containing protein (*ACRC*), and C-X-C motif chemokine receptor 3 (*CXCR3*); (**C**) Genetic organization of TAF1 and the approximate position of seven nucleotide variants. DSC, Disease-specific Single-nucleotide Changes; SVA, Short interspersed nuclear element; MTS, Multiple Transcript System. The variable number of tandem repeats, and Alu composite; del48, 48 bp deletion variant. Solid rectangles filled with black, *TAF1* exons; Solid rectangles filled with blue, MTS exons. (**D**) Exon structure of the human TAF1 cDNA. Positions of the start codon (ATG) and stop codon (TGA) are indicated. The size of the full-length coding region is described in the parenthesis. bp: base pairs. Neuron specific TAF1 transcript (N-TAF1) contains a full-length sequence derived from TAF1 exons and a sequence of six additional bp, named exon 34’ [44], is expressed exclusively in neurons, and is the second most abundant among all TAF1 species. A previous study demonstrated a marked reduction of N-TAF1 in caudate in a postmortem XDP brain [45].

Previous conventional linkage and association studies based on linkage disequilibrium have demonstrated that the XDP locus is within a 427 kb genetic interval on chromosome Xq13.1 [40] (Figure 1A). This region was recently further refined to 294 kb using whole genome sequencing [5]. The refined XDP haplotype includes four known genes: *TAF1*, *OGT*, *ACRC*, and *CXCR3* (Figure 1B). Seven nucleotide variants have been identified within the XDP haplotype: five disease-specific single-nucleotide changes (designated as Disease-specific Sequence Changes [DSC] 1, 2, 3, 10, and 12), one 48 bp deletion, and one 2627 bp SVA (SINE-VNTR-Alu element) retrotransposon insertion (Figure 1C). These variants are in complete linkage disequilibrium and no recombination events have been detected between the variants in 21 patients with XDP [5]. The variants are located either within the intronic region of *TAF1* or in a nonconventional exon, and the intronic region at the multiple transcript system mapped downstream of *TAF1*. Bioinformatics analysis indicated no supportive evidence that these variants are located within or near potential regulatory sequences. Copy number variants (CNVs) were investigated using whole-genome microarray analysis, but no disease-associated CNVs were detected in the candidate region for XDP [5]. Clinical and geographical analyses suggest that the disease-causing genetic defect is derived from a founder born in Panay Island in the Philippines, in whom a single de novo genetic defect occurred [41]. Because it is unlikely that two or more disease-associated variants will occur simultaneously in a single individual, one of the seven variants is most likely the cause of XDP pathology, whereas the other variants are simply linked changes that occurred on the same funder chromosome. Amplification of the inserted SVA variant using a long-range polymerase chain reaction has been used for genetic testing for XDP and has contributed to nationwide surveillance on XDP in the Philippines [42,43].

Although the exact disease-causing genetic variant remains to be elucidated, decreased expression levels of specific *TAF1* transcripts have been demonstrated in postmortem brain, blood, and fibroblasts obtained from patients with XDP [45,46]. This phenomenon was verified in induced pluripotent stem cell (iPSC)-derived neural stem cells (NSCs), suggesting that an aberrant transcription of *TAF1* occurs in neuronal cells at the early stages of development [47]. Moreover, *TAF1* dysfunction at the transcriptome level was also shown in peripheral biomaterials where 307 genes were dysregulated. A reduction of *TAF1* transcription would impair its biological role as an RNA polymerase II-dependent transcription factor, probably leading to the dysfunction of canonical genes, including synaptotagmin-like 2 (*SYTL2*) and solute carrier family 5 (inositol transporter), member 3 (*SLC5A3*) [46]. The gene *SYTL2* plays a biological role in membrane trafficking in peripheral secretory cells through interaction with RAS-associated protein 27A (RAB27A). The possible involvement of vesicle trafficking and synaptogenesis in XDP pathology was previously demonstrated in cultured cells expressing specific *TAF1* isoforms containing multiple transcript system (MTS) exons d3 and d4 [48]. *SLC5A3* regulates brain osmoregulation including inositol metabolism, which is impaired in the striatum in Huntington’s disease as well as cellular and animal models of the disease [49]. *TAF1* dysfunction and neurological disorder has also been demonstrated in another distinctive disease. Hemizygous missense and splice-site *TAF1* variants, and duplication involving *TAF1*, are associated with X-linked syndromic mental retardation 33 (OMIM 300966), which is a severe neurodevelopmental disorder and has dystonic features [50].

Considering the possible pathomechanisms caused by the reduced expression of specific *TAF1* transcripts, the SVA variant is in a position to lead to genetic predisposition to XDP. The SVA variant is inserted at intron 32 of *TAF1*, and might affect the expression of *TAF1* transcripts. Intriguingly, an SVA insertion has been reported to be active in the human genome and may cause disease in humans [51]. To date, two other diseases have been described wherein an SVA insertion is associated with a loss of mRNA expression: autosomal recessive hypercholesterolemia [52] and Fukuyama-type muscular dystrophy [53]. Genome editing technology [54] might provide more direct clues to help us determine the exact variant affecting *TAF1* transcription and function in iPSC-derived NSCs.

## 6. Functional Pathology

**Figure 2:**
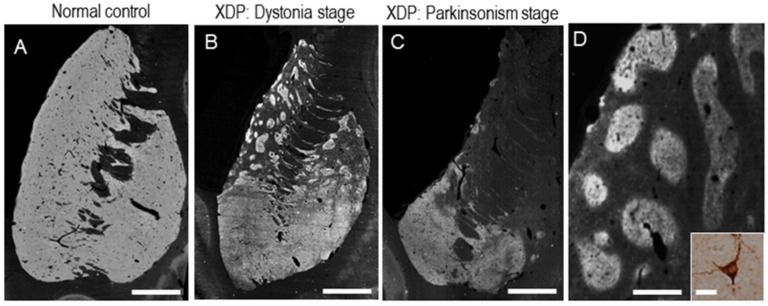
Striatal pathology of patients with X-linked dystonia-parkinsonism (XDP). Negative prints of the striatum were stained immunohistochemically for calcineurin, a marker for striatal medium spiny neurons, from a “normal” control (**A**) XDP patient at “dystonia” stage (**B**) and XDP patient at “parkinsonism” stage (**C**). (**D**) XDP patches visualized by calcineurin staining. The asterisk indicates an example of the XDP patches. A striatal neuron labelled for calcineurin is shown in the inset in (**D**). Scale bars: (**A**–**C**) = 4 mm; **D** = 1 mm; *inset* in **D** = 20 μm. CN = caudate nucleus; PUT = putamen; NA = nucleus accumbens. (From Goto, S. et al., Functional anatomy of the basal ganglia in X-linked recessive dystonia-parkinsonism. *Ann. Neurol.* 2005, 58, 7–17 Copyright 2005, with permission from Wiley InterScience; and Goto, S. et al. Defects in the striatal neuropeptide Y system in X-linked dystonia-parkinsonism. *Brain.* 2013; 136 (Pt 5): 1555–1567 Copyright 2013, with permission from Oxford University Press).

Progressive neuronal loss with replacing astrogliosis in the neostriatum (i.e., putamen and caudate nucleus) has so far been identified as the primary neuropathology of XDP [8,9]. Postmortem studies on patients with XDP have shown a clear correlation between clinical manifestation and striatal pathology [8,9]. As a clinical sign, dystonia is the leading symptom in the early disease stage (“dystonia” stage), but is associated with or replaced by parkinsonism in the later years of life (“parkinsonism” stage) [3]. As shown in Figure 2, at the “dystonia” stage (Figure 2B,D), striatal pathology is characterized by a compartmental loss of medium spiny neurons (MSNs) in the dorsal striatum, where the striosome compartment is severely depleted but the matrix compartment is relatively spared [8,9]. This compartment-specific loss of MSNs results in a striatal mosaic composed of multiple patches of remaining matrix MSNs. These findings raise the possibility that dystonia is a feature indicative of striosomal dysfunction. In addition, eventual loss of the direct pathway neurons also may cause hyperkinetic symptoms in XDP. In contrast, in the parkinsonian phase (Figure 2C), there is a greater loss of MSNs in both the striosome and matrix compartments, which leads to a marked reduction in the interactions of their fibers with the globus pallidus and other extrastriatal brain regions [8,55]. There is a sparing of cholinergic striatal interneurons and a significant loss of cells positive for neuropeptide Y (NPY) in patients with XDP [8,9]. 

**Figure 3:**
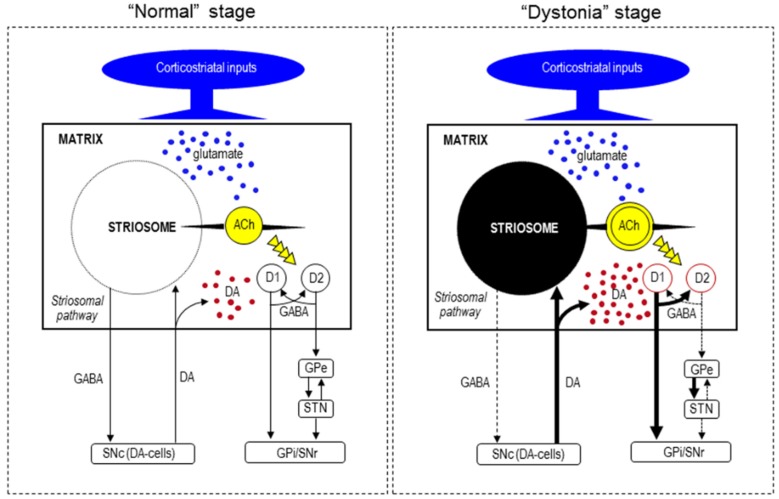
Hypothesized diagram for functional anatomy of the basal ganglia in XDP. At the “dystonia” stage, the severe loss of the striosomal pathway might lead to the disinhibition of DA-cells in the SNc, thereby increasing the action of striatal dopamine on the remaining matrix MSNs. This might result in overactivation of the basal ganglia-thalamocortical circuit, which leads to hyperkinetic symptoms such as dystonia. In addition, loss of striosomal cells may cause an overactivation of cholinergic interneurons. Abbreviations: SNc, substantia nigra pars compacta; DA-cells, dopamine-producing cells; D1, dopamine D1 receptor; D2, dopamine D2 receptor; GPe, globus pallidus externa; STN, subthalamic nucleus; GPi, globus pallidus internus; SNr, substantia nigra pars reticulata; ACh, acetylcholine.

Striosomal MSNs send their GABAergic projections directly to the substantia nigra pars compacta (SNc), which contains dopamine-producing cells (DA-cells) that project back to both the striosome and matrix compartments [56,57]. Thus, the striosome compartment is in a position to exert global control over dopamine signaling in the striatum. The striosome compartment is also thought to communicate with the matrix compartment via striatal local-circuit interneurons [56,57,58]. Among these interneurons, cholinergic cells are particularly interesting, as they can control dopamine release and their dysfunction is thought to be involved in the genesis of dystonia [59]. Moreover, a computational model of the cortico-basal ganglia circuit has shown that the striosome compartment plays a critical role in modular reinforcement learning [60], which could participate in the brain function of motor “selection” or “focusing”. Based on the functional model of basal ganglia in XDP [8], we hypothesize that at the earlier “dystonia” stage, the severe loss of the striosomal pathway leads to disinhibition of DA-cells in the SNc, thereby increasing the action of striatal dopamine on the remaining matrix MSNs (see Figure 3). This might result in overactivation of the basal ganglia-thalamocortical circuit, which leads to the hyperkinetic symptoms of dystonia. A neurophysiological study [20] has shown that the dysfunction of interconnections between the anterior cingulate cortex and the striatum, which are known to favor the striosome compartment, might affect error-related behavioral adaptation in XDP patients with dystonia.

Our assumption that an increase in dopaminergic activation in the remaining MSNs might occur in the striatum in XDP is supported by the PET finding that patients with XDP and generalized dystonia have striatal rate constants for [^18^F] fluorodopa uptake that are in the normal range [25] despite, as shown here, a severe loss of striatal cells. At the later “parkinsonism” stage, the greater involvement of the matrix compartment leads to a severe and critical reduction in matrix-based projections and thus to the development of a so-called “extranigral form” of parkinsonism. This condition has also been suggested in the late (or end) stage of HD [61]. Nuclear imaging studies have also suggested that parkinsonism in XDP might result from post-synaptic striatal dysfunction [28].

## 7. Pathogenesis of Striatal Neurodegeneration

The cellular and molecular processes that lead to the neurodegeneration that occurs selectively in the neostriatum at maturity in XDP have not been elucidated to date. The gene *TAF1* is ubiquitously expressed and is an essential component of the multi-subunit transcription factor IID (TFIID) complex, which is involved in transcription initiation by RNA polymerase II [6]. As TFIID plays a critical role in general transcription in living cells, it is thought that the disruption of *TAF1* function potentially has broad effects on gene expression. An XDP-specific haplotype has been identified as consisting of seven sequence variants clustering around the human *TAF1* gene [4,45,46]. These variants include five single nucleotide substitutions designated DSC 1, 2, 3, 10, and 12, a 48 bp deletion, and an SVA-type retrotransposon insertion. A direct or specific link between these variants and disease pathogenesis has not been clarified, and there seems to be a diversity of molecular and cellular factors that contribute to the striatal pathology in XDP, as has also been suggested in HD [62]. 

Domingo et al. [46] used peripheral models of XDP (i.e., blood and fibroblasts) to show that consistent dysregulation of common *TAF1* transcripts was often accompanied by altered genome-wide gene expression, along with an enrichment of genes and networks related to transcriptional processes. Given the possible link between XDP-specific genetic alterations and the dysfunction of a canonical gene, the authors suggested that *TAF1* dysregulation may drive changes in gene expression in the brains of XDP-affected individuals. Given that nuclear factor-kappa B (NFκB) signaling is present in both fibroblasts and neural stem cells from patients with XDP, Vaine et al. [63] suggested that the transcription pathway of NFκB may be a site of dysfunction in XDP. Herzfeld et al. [48] described a pathological role for DSC3 in XDP because DSC3 is located within exon d4 of the *TAF1/DYT3* multiple transcript system, even though other sequence changes are intronic. Using neuroblastoma cells, the authors showed an effect of DSC3 on broad gene expression accompanied by an enrichment of genes related to vesicle and synaptic function, dopamine metabolism, Ca^2+^ metabolism, and protection against oxidative stress. We have suggested that the neostriatal pathology in XDP might be associated with the impaired expression of NPY [9], which is a novel bioactive substance with a role in the modulation of neurogenesis and neurotransmitter release, and thereby exerts a protective influence against neurodegeneration [64]. We performed a postmortem analysis to determine the striatal localization profile of NPY in normal individuals and in patients with XDP [9]. In normal controls, we found a scattered distribution of NPY-positive neurons and numerous nerve fibers labeled for NPY in the striatum, where there was a differential localization of NPY immunoreactivity in the striatal compartments, and a heightened density of NPY labeling in the matrix compartment relative to the striosomes [9]. In patients with XDP, there was a significant decrease in the number of NPY-positive cells accompanied by a marked loss of their nerve fibers in the neostriatum, and a lack of NPY labeling in the subventricular zone with a marked loss of progenitor cells [9]. These findings suggest the presence of a neostriatal defect in the NPY system in patients with XDP. Therefore, the NPY system may be implicated in the mechanism by which a compartmental loss of striatal neurons progressively occurs in XDP.

## 8. Conclusions

XDP typically affects adult male Filipinos whose ancestries originate from the Philippine island of Panay. XDP is unique among movement disorders, as it leads to progressive and severe dystonia in the earlier disease stages, which are followed by overt parkinsonism in the later stages. In accordance with this clinical course, postmortem neuropathological analyses have shown that striatal neurons progressively degenerate in a cell-type and compartment-specific manner. Genetic analyses have shown that XDP is a transcriptional dysregulation syndrome caused by impaired expression of the *TAF1* gene. An XDP-specific haplotype has been identified and consists of seven sequence variants clustering around the human *TAF1* gene, although a direct link of these variants with disease pathogenesis has not yet been established. In addition to striatal deregulation of dopamine transmission, multiple molecular and cellular factors have been suggested to contribute to the development of striatal symptomatology and pathology in XDP, similar to what has so far been described in HD [65]. A further study on the specific link between the DYT3-specific impairment of the *TAF1* gene and striatal cell vulnerability would be required to determine the precise pathomechanism by which progressive neurodegeneration occurs selectively in the neostriatum at maturity in XDP.
